# Radiation-induced organizing pneumonia after stereotactic body radiotherapy for lung tumor

**DOI:** 10.1093/jrr/rrv049

**Published:** 2015-09-02

**Authors:** Satoru Ochiai, Yoshihito Nomoto, Yasufumi Yamashita, Shuuichi Murashima, Daisuke Hasegawa, Yusuke Kurobe, Yutaka Toyomasu, Tomoko Kawamura, Akinori Takada, Noriko II

**Affiliations:** 1 Department of Radiation Oncology, Matsusaka Central Hospital, 102 Kobou Kawai-machi, Matsusaka, Mie, 515-8566, Japan; 2 Department of Radiology, Mie University School of Medicine, 2-174 Edobashi, Tsu, Mie 514-8507, Japan; 3 Department of Radiology, Matsusaka Central Hospital, 102 Kobou Kawai-machi, Matsusaka, Mie, 515-8566, Japan

**Keywords:** organizing pneumonia, BOOP syndrome, radiation pneumonitis, stereotactic body radiotherapy, stereotactic ablative radiotherapy, lung cancer, lung tumor

## Abstract

The aim of this retrospective study was to investigate characteristics of organizing pneumonia (OP) after stereotactic body radiotherapy (SBRT) for lung tumor. Between September 2010 and June 2014, patients who were diagnosed as Stage I lung cancer and treated with SBRT at our institution were included in this study. A total of 78 patients (47 males with a median age of 80 years) were analyzed. The median follow-up period was 23 months. Five patients (6.4%) developed OP at 6–18 months after SBRT. The cumulative incidence of OP was 4.3% (95% confidence interval [CI], 1.1–11.0) and 8.2% (95% CI, 2.9–17.0) at 1 and 2 years, respectively. Tumor location (superior and middle lobe vs inferior lobe) was shown to be a borderline significant factor for the occurrence of OP (***P*** = 0.069). In the subgroup analysis of patients with a radiographic follow-up period at least 6 months, or who died within 6 months after SBRT, 7 of 72 patients (9.7%) developed Grade 2 or 3 radiation pneumonitis (G2/3 RP) at 2–4 months after SBRT. A statistically significant association between G2/3 RP in the subacute phase and OP was shown (***P*** = 0.040). In two of the five patients who developed OP, the symptoms and radiographic change were improved rapidly by corticosteroid administration. One patient had relapsed OP after suspending the treatment and re-administration was required. Three patients with minor symptoms were managed without corticosteroid administration and OP resolved without any relapse. The radiation-induced OP should be considered as one of the late lung injuries after SBRT for lung tumors.

## INTRODUCTION

Stereotactic body radiotherapy (SBRT), also called stereotactic ablative radiotherapy (SABR), has been widely used as an effective and safe treatment for early-stage lung cancer, especially in medically inoperable cases [1, 2]. SBRT provides a favorable local control and survival benefit compared with conventional radiotherapy [1–3]. Severe clinical toxicities after SBRT are fairly uncommon for the treatment of peripheral lung tumors, and the rate of Grade 5 complications was reported as 0.6% in the nationwide survey in Japan [4].

Radiation pneumonitis (RP) is one of the most common toxicities after SBRT for lung tumors [1 , 2, 5]. Although a few cases were severe and there was a risk of mortality [4, 6, 7], most of the RP was Grade 1 or 2 and either asymptomatic or manageable. The rate of symptomatic RP is typically <10% [5].

Radiation-induced organizing pneumonia (OP), also called bronchiolitis obliterans organizing pneumonia (BOOP), is another lung injury that occurs after thoracic radiotherapy [8–17]. There are several differences between OP and RP. (i) RP occurs during or shortly after the completion of radiotherapy, while OP occurs several months after the completion of radiotherapy; (ii) RP lesions are limited to the irradiated area, while OP lesions are characterized by lung infiltrates outside the radiation field and frequently migrate; (iii) RP always results in fibrosis and never relapses, while OP usually resolves without fibrosis but commonly relapses when the corticosteroid is withdrawn [14, 15].

Although OP after post-operative radiotherapy (PORT) for breast cancer has been investigated in numerous studies [8–14], there are few reports describing OP after SBRT for lung tumors [16, 17]. This retrospective study was conducted to investigate the characteristics of OP after SBRT for early-stage lung cancer.

## MATERIALS AND METHODS

### Study design and patient selection

This single-institutional retrospective study was conducted after approval by our institutional review boards. The necessity for informed consent for inclusion in this study was waived.

Between October 2010 and June 2014, 88 patients with a solitary lung tumor were treated with SBRT at our institutions. Among them, 78 (88.6%) patients had T1N0M0 or T2aN0M0 stage lung cancer according to the 7th edition of UICC TNM staging and were included in this study. They all had a solitary lung tumor of <5 cm, with no evidence of metastases in pretreatment evaluation. Patients who had a tumor that was not confirmed by histology but who had a significant uptake of 18F-fluorodeoxyglucose (FDG) on positron emission tomography (PET) and were diagnosed clinically as having lung cancer by pulmonologists and radiologists were included in this study. Patients who had undergone curative resection of another non-small cell lung cancer (NSCLC) were also included if the target lung tumor was in a lung lobe different from the one that had been treated previously.

Pretreatment evaluation was performed by a routine physical examination, with laboratory tests and imaging studies, including chest radiograph, chest and abdominal computed tomography (CT) with or without contrast-enhanced medium, brain magnetic resonance imaging and FDG PET/CT studies. After January 2011, serum Krebs von den Lungen (KL)-6 level was evaluated routinely, and the pretreatment KL-6 level was available in 71 of 78 (91.0%) patients. The characteristics of evaluated patients are shown in Table 1.

**Table 1.** The characteristics of patients

Variables		No. of patients (%)
Age	median age (years) (range)	80 (46–91)
Sex	male	47 (60.3)
	female	31 (39.7)
Emphysematous change in lung	yes	39 (50.0)
	no	39 (50.0)
Serum KL-6 level^a^	median (U/ml) (range)	285 (109–711)
Tumor size	median (mm) (range)	22 (8–45)
Tumor location in lung	superior or middle lobe	46 (61.5)
	inferior lobe	30 (38.5)

^a^Pretreatment serum KL-6 was available in 71 (91.0%) patients. KL-6 = Krebs von den Lungen-6.

### SBRT

Our methods for treatment planning have been described in detail previously [18]. The dose fractionation schedule and SBRT parameters are shown in Table 2. SBRT plans were generated using the Pinnacle planning system (Phillips Medical System, Andover, MA). Monitor units were calculated using a collapsed cone convolution algorithm. The clinical target volume was defined as the visible gross tumor volume. The internal target volume (ITV) was chosen considering CT using a slow scan technique. The planning target volume (PTV) was defined as the ITV with a 5-mm margin to allow for set-up uncertainty. A respiration-monitoring apparatus was used for breath-holding conditions if the amplitude of tumor motion was large. In the case of the amplitude of tumor motion being within 1.5 cm in each direction, instead of using the breath-holding technique, the range of the tumor was considered in delineation of ITV. The dose was prescribed to the isocenter. A total dose of 40–60 Gy was administered in 4–10 fractions. We typically prescribed a total dose of 48 Gy in 4 fractions. A prescription of total dose of 52–54 Gy in 4 fractions or 60 Gy in 6 fractions was used in some patients with tumors >3 cm, and of 40 Gy in 4 fractions or 60 Gy in 8–10 fractions in patients who had tumors near critical organs such as the brachial plexus, esophagus or great vessels. The overall treatment time of SBRT ranged from 4 to 12 days in cases of 4 fractions and from 9 to 18 days in cases of 6–10 fractions. The total lung volume covered with 20 Gy or more (Lung V20) and the mean lung dose (MLD) were evaluated on the treatment planning workstation. Irradiation was performed using 6-MV photons from an Elekta Synergy linear accelerator (Elekta Inc., Peachtree, GA) in coplanar and non-coplanar static ports (7 to 13 ports). Daily online cone-beam CT-based volumetric image-guided radiation therapy using soft tissue target registration was applied immediately prior to any SBRT.

**Table 2.** Dose fractionation and SBRT parameters

Dose fractionation, Gy/Fractions	No. of patients (%)
40/4	2 (2.6)
48/4	58 (74.4)
52/4	3 (3.8)
54/4	1 (1.3)
60/6	2 (2.6)
60/8	11 (14.1)
60/10	1 (1.3)
Median total dose (Gy) (range)	48 (40–60)
Median dose per fraction (Gy) (range)	12.0 (6.0–13.5)
Median number of fractions (range)	4 (4–10)
Median overall treatment time (days) (range)	5 (4–18)
Median lung V20 (%) (range)	6.3 (2.4–14.7)
Median mean lung dose (Gy) (range)	4.0 (2.1–10.1)

Lung V20 = lung volume covered with 20 Gy or more.

### Diagnosis of OP after SBRT

The criteria for the diagnosis of OP after SBRT were defined by reference to previous criteria for SBRT of the lung and PORT for breast cancer [8–16]: (i) a mixture of patchy and ground-glass opacity, (ii) general and/or respiratory symptoms lasting for at least 2 weeks, (iii) synchronous or metachronous (migrated) radiographic lesion in the lung volume receiving <0.5 Gy, and (iv) no evidence of a specific cause. The diagnosis was determined by three radiation oncologists (S.O., Y.Y. and Y.N.) and three radiologists (S.M., D.H. and Y.K).

### Follow-up studies

Patients were followed until February 2015 or death. CT scans were performed every 3 months until 2 years after the treatment and were repeated every 4–6 months thereafter. Toxicities of SBRT were evaluated using the Common Terminology Criteria for Adverse Events Version 4. The follow-up period after SBRT ranged from 0 to 63 months (median, 23 months).

### Statistical analysis

The main endpoint of this study was the incidence of OP after SBRT. The endpoint was calculated from the first day of SBRT. The cumulative incidence of OP was calculated by accounting for death as a competing risk [19, 20]. To investigate the risk factor for development of OP, the incidence of OP was first compared using Gray's test between the following covariates: age, sex, pretreatment serum KL-6 level, prior surgery for another lung cancer, tumor size, emphysematous change in lung, tumor location, total dose of SBRT, dose per fraction, number of fractions, overall treatment time, MLD and Lung V20 [21]. The threshold values for Gray's test were medians in variables. The threshold of the serum KL-6 level was also selected as the median in this study. Although the cut-off level in the diagnosis of interstitial pneumonia is 500 U/ml, the optimal cut-off level as a predictor of lung injury after radiation therapy is still under debate [22]. Spearman's rank correlation coefficient was used to evaluate the relationship between variables.

Second, we conducted a subgroup analysis of patients with a radiologic follow-up period at least 6 months or who died within 6 months after SBRT (72/78, 92.3%) to evaluate the OP as a late adverse event after SBRT. In this analysis, the cumulative incidence of Grade 2 or 3 RP (G2/3 RP) was also calculated. We evaluated the impact of G2/3 RP within 6 months (subacute phase) on the occurrence of OP with a landmark method [16, 23].

A *P*-value of <0.05 was inferred as statistically significant. All statistical analyses were performed using EZR (Saitama Medical Center, Jichi Medical University, The R Foundation for Statistical Computing) [24].

## RESULTS

### The incidence of OP

**Fig. 1.**
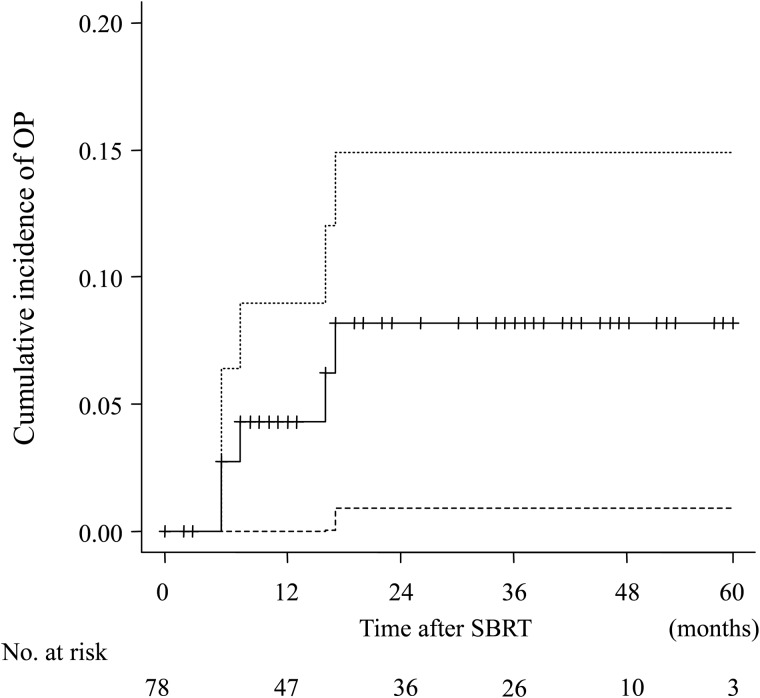
Cumulative incidence of organizing pneumonia (OP) after stereotactic body radiotherapy (SBRT) for lung tumor. The dashed lines represent the 95% confidence intervals. The cumulative incidence of OP was 4.3% (95% confidence interval (CI), 1.1–11.0) and 8.2% (95% CI, 2.9–17.0) at 1 year and 2 years, respectively.

Five patients developed OP after SBRT during the follow-up and the crude incidence was 6.4% (5/78). The development of OP was observed at 6–18 months after SBRT. Of 78 patients, 10 (12.8%) had died by the end of follow-up. The cumulative incidence of OP was 4.3% (95% confidence interval (CI): 1.1–11.0) and 8.2% (95% CI: 2.9–17.0) at 1 year and 2 years, respectively (Fig. 1).

### Clinical course and characteristics of OP patients

**Fig. 2.**
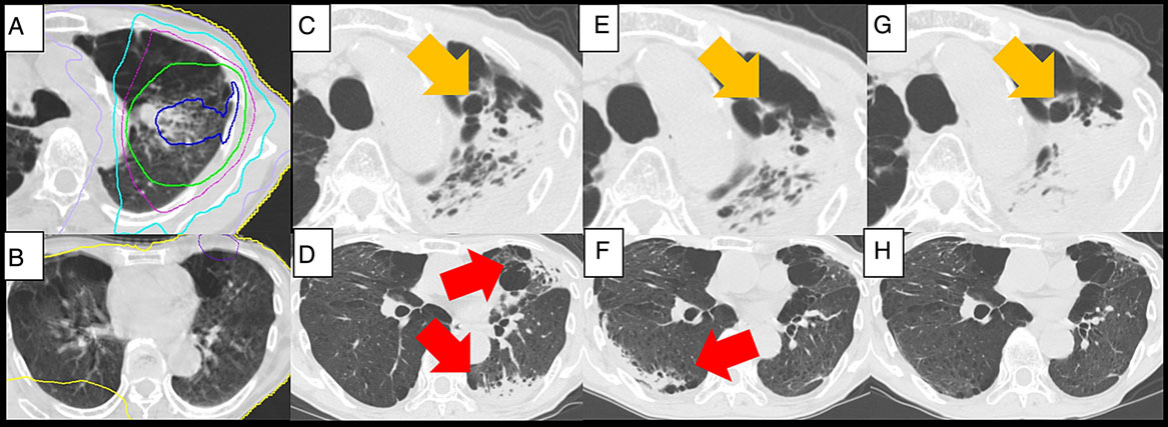
Chest CT and dose distribution of stereotactic body radiotherapy (SBRT) of a 70-year-old male patient. Dose distribution of SBRT. Isodose lines of 60 Gy (blue), 50 Gy (green), 40 Gy (purple), 30 Gy (light blue), 20 Gy (lavender) and 0.5 Gy (yellow) (A, B). Radiation pneumonitis (RP) represents in high-dose irradiated area (**C**, orange arrow) and patchy opacity in left lung appeared 6 months after SBRT (**D**, red arrow). No significant change in RP 9 months after SBRT (**E**, orange arrow). The radiographic lesion in the left lung disappeared and a new lesion in the right lung appeared. (F, red arrow). These radiographic changes were considered to be the sequential change of organizing pneumonia (OP) (migration). RP resulted in fibrosis (G, orange arrow), and OP resolved without fibrosis at 12 months after SBRT (H).

The clinical course and characteristics of OP are summarized in Table 3. An example of OP is shown in Fig. 2. Four males and one female developed OP, and the age ranged from 70 to 85 years old. The irradiated dose was 48 Gy in four fractions in three patients and 60 Gy in eight fractions in another two patients. G2/3 RP was observed in two patients before the development of OP. Two patients had major (Grade 2) symptoms. They were administered with corticosteroid as an initial treatment for OP, and symptoms and radiographic change improved rapidly. Relapse of OP was observed in one patient after suspending corticosteroid administration, and re-administration of corticosteroid was required. Corticosteroids to the two patients were being tapered at the time of the analysis. The other three patients with minor symptoms (Grade 1) were managed without corticosteroid treatment and the symptoms and the radiographic change were resolved within 3–6 months after diagnosis. No relapse was observed in these patients during the follow-up.

**Table 3.** The summary of clinical characteristics and course of OP

No.	Sex	Age	Dose/Fx	RP	Symptoms of OP				Tx	Clinical course
					Fever	Fatigue	Cough	Dyspnea		
1	M	74	48/4	Yes	0	1	1	1	Obs	Resolved
2	M	70	60/8	No	1	1	0	1	Obs	Resolved
3	M	85	48/4	Yes	1	2	1	2	PSL	Relapse after remission
4	M	79	60/8	No	1	2	0	2	PSL + Abx	Remission
5	F	74	48/4	No	0	0	1	1	Abx	Resolved

Fx = fractions, RP = Grade 2 or 3 radiation pneumonitis, OP = organizing pneumonia, Tx = treatment, M = male, F = female, Obs = observation, PSL = prednisolone, Abx = antibiotics.

### Risk factor analysis of OP after SBRT

The result of Gray's test is summarized in Table 4. No statistically significant factor for occurrence of OP was detected in this analysis. Tumor location (superior/middle lobe vs inferior lobe) was shown to be a borderline statistically significant factor (*P* = 0.069). There was no significant correlation between tumor location and other variables. The number of fractions (4 fractions vs >4 fractions) had a trend toward significance (*P* = 0.112). There were very strong correlations between number of fractions and total dose (*ρ* = 0.854, *P* < 0.001) and dose per fraction (*ρ* = –0.864, *P* < 0.001). There was moderate correlation between number of fractions and overall treatment time (*ρ* = 0.576, *P* < 0.001). Although OP was not observed in patients with prior surgery for another cancer, this factor was not statistically significant (*P* = 0.215).

**Table 4.** The results of Gray's test

Variables		No. of patients (%)	2-year cumulative incidence of OP	*P* value
Age	≤80 years	40 (51.3)	11.5	0.288
	>80 years	38 (48.7)	3.6	
Sex	Male	47 (60.3)	12.7	0.259
	Female	31 (39.7)	3.3	
Prior surgery	Yes	19 (24.4)	0.0	0.215
	No	59 (75.6)	10.5	
serum KL-6 level^a^	≤285 U/ml	35 (49.3)	6.3	0.575
	>285 U/ml	36 (50.7)	12.8	
Emphysematous change	Yes	39 (50.0)	8.3	0.667
	No	39 (50.0)	8.6	
Tumor size	≤22 mm	37 (47.4)	5.9	0.688
	>22 mm	41 (52.6)	11.2	
Tumor location	Sup or mid	48 (61.5)	13.4	0.069
	Inf	30 (38.5)	0.0	
Total dose	≤48 Gy	60 (76.9)	6.0	0.202
	>48 Gy	18 (23.1)	17.3	
Dose per fraction	<12 Gy	16 (20.5)	16.8	0.232
	≥12 Gy	62 (79.5)	6.0	
Number of fractions	4	64 (82.1)	5.8	0.112
	>4	14 (17.9)	20.5	
Overall treatment time	≤5 days	47 (60.3)	7.6	0.809
	>5 days	31 (39.7)	9.9	
Mean lung dose	≤4.0 Gy	39 (50.0)	6.5	0.661
	>4.0 Gy	39 (50.0)	9.7	
Lung V20	≤6.2%	39 (50.0)	6.4	0.622
	>6.2%	39 (50.0)	9.9	

OP = organizing pneumonia, Prior surgery = prior surgery for another lung cancer, KL-6 = Krebs von den Lungen-6, Sup = Superior lobe, Mid = middle lobe, Inf = inferior lobe, Lung V20 = lung volume covered with 20 Gy or more. ^a^Pretreatment serum KL-6 level was available in 71 (91.0%) patients.

### Relationship between G2/3 RP in subacute phase and OP

**Fig. 3.**
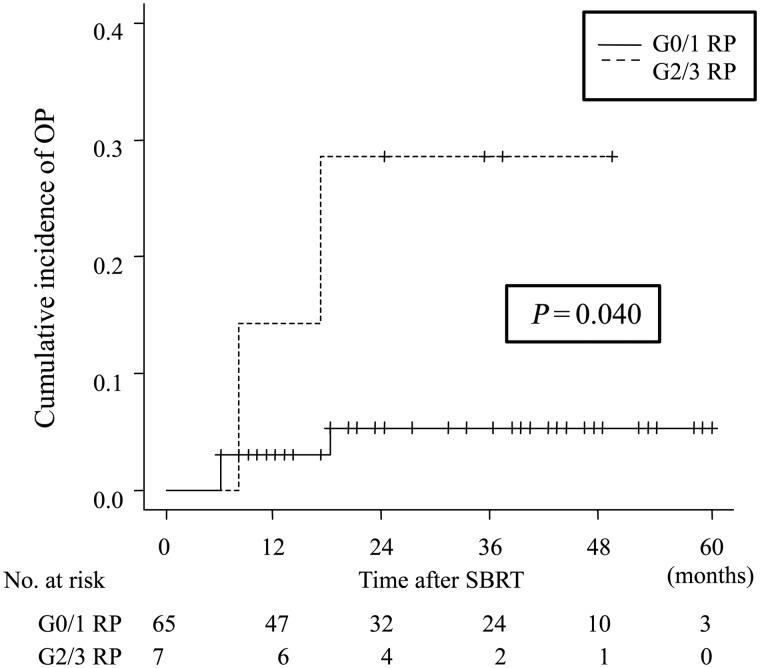
Comparison of the cumulative incidence of organizing pneumonia (OP) of patients with or without Grade 2 or 3 radiation pneumonitis (G2/3 RP) within the subacute phase with landmark method. G2/3 RP 2 in subacute phase was shown to be a significant risk factor for development of OP, compared with those without G2/3 RP (Gray's test *P* = 0.040).

Of the 78 patients, 72 (92.3%) were followed up radiologically for at least 6 months or died within 6 months after SBRT. These patients were analyzed for OP as a late adverse event after SBRT. Among them, 7 patients (9.6%) developed Grade 2/3 OP after SBRT. Of these, Grade 3 RP occurred in 1 patient and the others had Grade 2 RP. G2/3 RP developed at 2–4 months (subacute phase) after SBRT, and the cumulative incidence of G2/3 RP was 9.7% (95% CI, 4.2–17.9) at 6 months. No statistically significant factor for developing G2/3 RP was found in Gray's test in this subgroup. G2/3 RP in the subacute phase was shown to be a statistically significant risk factor for development of OP, using a landmark method (*P* = 0.040) (Fig. 3). The 1-year and 2-year cumulative incidence was 14.3% and 28.6% in patients with prior G2/3 RP and 3.1% and 5.4% in patients without G2/3 RP, respectively (Fig. 3).

## DISCUSSION

In this study, we retrospectively analyzed the OP after SBRT for early-stage lung cancer and described the characteristics of OP. Although numerous studies have been reported for OP after PORT for breast cancer [8–15], there are few reports describing OP after SBRT for lung tumors [16, 17]. Table 5 summarizes the reported results of OP after PORT for breast cancer and after SBRT of the lung.

**Table 5.** Reported results of OP after PORT for breast cancer and SBRT.

Series	Material	No. of Pts	Crude incidence (%)	Latency period range (months)	Related factor
Takigawa *et al*. [10]	PORT for breast cancer	157	2.5	5–6	Immunologic reaction
Miwa *et al*. [25]	PORT for breast cancer	206	2.4	2–7	Not evaluated
Ogo *et al*. [12]	PORT for breast cancer	2056	2.4	0–10.5 (mean 4.2)	No significant factor
Katayama *et al*. [13]	PORT for breast cancer	702	2.3	2.3–7.9 (median 3.8)	Age >50 Endocrine therapy Irradiated lung volume
Ogo *et al*. [11]	PORT for breast cancer	616	1.9	3–12 (mean 5.6)	No significant factor
Oie *et al*. [14]	PORT for breast cancer	428	1.2	5.6–7.6 (median 7.3)	RP
Murai *et al*. [16]	SBRT	189	4.8	6–16	Symptomatic RP
This study	SBRT	78	6.4	6–18	Grade 2 or 3 RP (Superior lobe of lung)

PORT = postoperative radiotherapy, SBRT = stereotactic body radiotherapy, RP = radiation pneumonitis.

In our series, OP occurred in 5 out of 78 (6.4%) patients after SBRT, and the cumulative incidence of OP was 4.3% and 8.2% at 1 year and 2 years, respectively. Murai *et al*. reported OP after SBRT for primary or metastatic lung cancer [16]. They reported that the crude incidence of OP was 4.8% (9/189) and the cumulative incidence was 4.0% and 5.2% at 1 year and 2 years, respectively. Our incidence of OP was similar to their results. The incidence of OP after PORT for breast cancer has been reported to be ∼2% [8–15]. The incidence of OP after SBRT of the lung seemed to be high compared with that after PORT for breast cancer.

The interval from SBRT to development of OP ranged from 6 to 18 months in our study. Murai *et al*. reported that the interval ranged from 6 to 16 months [16]. Our results were quite similar to theirs. Takeda *et al*. also reported one case of OP in their prospective trial of SBRT, and it occurred at 6 months after SBRT [17]. It has been reported that most occurrences of OP after breast irradiation develop within 6 months [8–15]. The latency period for development of OP after SBRT is relatively long compared with that of PORT for breast cancer. This might be affected by the differing lengths of overall treatment time.

There are several differences between OP and RP. (i) RP occurs during or shortly after the completion of radiotherapy, whereas OP occurs several months after completion of radiotherapy; (ii) RP lesions are limited to the irradiated area, whereas OP lesions are characterized by lung infiltrates outside the radiation field and frequently migrate; (iii) RP always results in fibrosis and never relapses, whereas OP usually resolves without fibrosis but commonly relapses when the corticosteroid is withdrawn [14, 15]. While RP is known to be a direct effect of irradiation, the underlying mechanism that develops radiation-induced OP remains unclear. An autoimmune process is assumed to be at the base of the development of OP. Irradiation provokes tissue damage via sensitization of autoreactive lymphocytes, which react with pulmonary tissue [25]. Thus, OP is clearly different from RP. However, it has been reported that there is close relationship between RP and OP after PORT for breast cancer [12, 14]. Murai *et al*. reported that most OP occurred a few months after RP, and that there was a very strong association between prior symptomatic RP and OP after SBRT of the lung [16]. We also found a statistically significant correlation between G2/3 RP and the development of OP in the subgroup analysis of patients with at least 6 months of follow-up period. Our results support their findings, and there could be a close relationship between OP and RP after SBRT for lung tumor as well as PORT for breast cancer. We evaluated MLD and Lung V20 as parameters for risk assessment of RP during treatment planning. After January 2010, pre-treatment KL-6 was routinely evaluated. Although these have been reported as predictive factors for the development of RP [5, 21, 26–28], no direct relationship between these factors and OP was shown in this analysis.

**Fig. 4.**
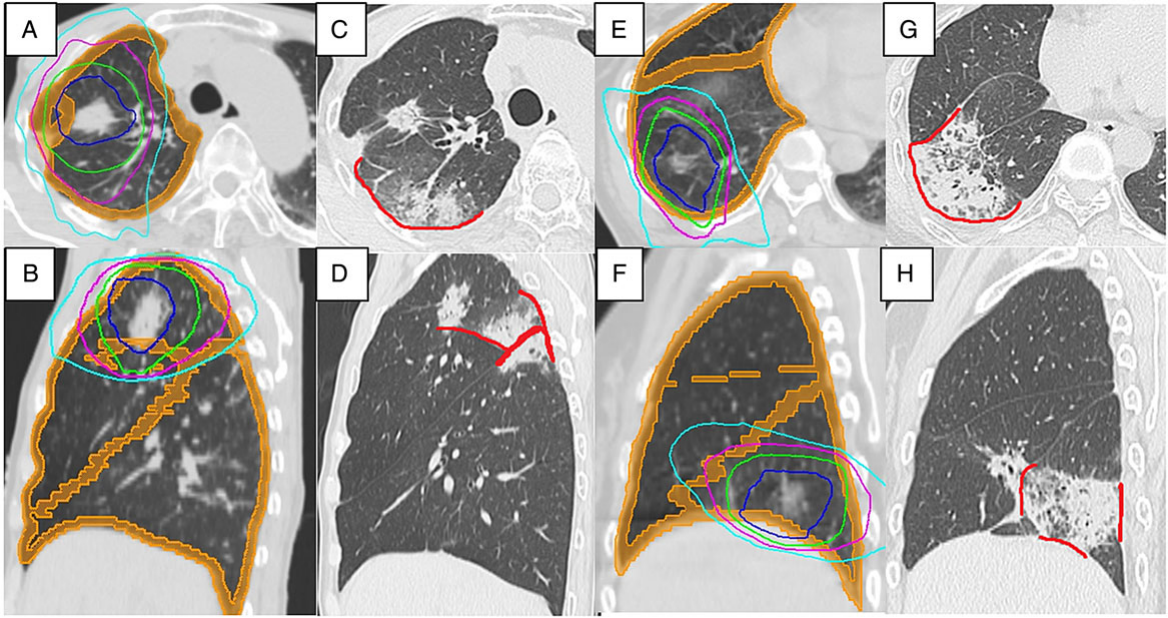
Dose distribution of stereotactic body radiotherapy (SBRT) for a tumor in superior (**A, B**) and in inferior lobe (**E, F**). Prescribed doses were both 48 Gy in four fractions. Isodose lines of 45 Gy (blue), 40 Gy (green), 30 Gy (purple) and 20 Gy (light blue) are shown. Orange regions represent subpleural region of the lung or pleura. Chest computed tomography (CT) scans at 3 months after SBRT are shown (**C, D, G, H**). The red lines represent the lesions where the radiation pneumonitis (RP) attached to the pleura. Both patients developed symptomatic RP. Compared with the patient with a tumor in the inferior lobe (G, H), the RP lesion attached more extensively to the pleura in the patient with a tumor in the upper lobe (C, D). The patient with a tumor in the upper lobe developed OP 6 months after SBRT. The RP of another patient with a tumor in the lower lobe was resolved without any relapse.

In our series, tumor location (superior/middle lobe vs inferior lobe) was shown to be a borderline statistically significant factor. Indeed, only patients with tumors in the superior lobe developed OP in our cohort. This result might indicate that the region where the RP occurs is the factor for developing OP. The pleural factor was indicated as the key to understanding the development of OP after PORT for breast cancer in some reports [8, 29]. With PORT for early-breast cancer, tangential fields are used to limit the dose given to the lung in order to reduce the incidence of RP. Tangential fields mainly induce an irradiation of subpleural regions of the lung. Crestani *et al*. reported that infiltrates of OP after PORT for breast cancer began in the irradiated area then spread to non-irradiated areas of the ipsilateral lung [8]. Oie *et al* reported an imaging study with CT and that most OP lesions developed in close proximity to the RP lesions [14]. According to these findings, it seems that the damage to subpleural region of the lung and pleura could be the primer for OP. In SBRT, lung tumor is irradiated with multiple non-coplanar beams in fewer fractions. As a result, relatively extensive subpleural region of lung or pleura could be irradiated by a high biological effective dose just like PORT for breast cancer. 50 Gy in 25 fractions approximately equivalent to 26 Gy in 4 fracions or 38 Gy in 8 fractions using the linear quadratic model, with an alpha/beta ratio of 3 Gy (Fig. 4).

The number of fractions (4 fractions vs >4 fractions) also had a trend toward significance in our analysis. However, this was considered to be a confounding factor. Tumors near the brachial plexus tended to be irradiated in larger number of fractions at our institution.

The optimal management of radiation-induced OP is still controversial [8–16, 30–32]. OP may be monitored if symptoms are minimal [32]. The effectiveness of macrolide antibiotics for OP has been reported [11, 30–32]. If OP worsens, corticosteroid administration can be applied. It provides rapid improvement of symptoms and radiographic change in most patients. Although relapse of OP is not rare after suspending the treatment or while the corticosteroids are tapered, administration of the least amount for the shortest possible period of time is preferable [30–32].

Two patients who developed OP with major symptoms were treated with corticosteroids as the initial treatment for OP in our series. Symptoms and radiographic change improved rapidly in these patients. However, OP relapsed in one patient after suspending administration of corticosteroid and re-administration was required. The other three patients, with minor symptoms, were managed without corticosteroid treatment. The symptoms and the radiographic change in these patients were resolved within 6 months and no relapse was observed during the follow-up. These results were similar to OP after PORT for breast cancer and indicated that the management of OP after PORT for breast cancer could be applied to OP after SBRT for lung tumors.

The retrospective nature of this study is an important study limitation. Lack of histological evaluation also poses a limitation. The diagnoses of OP were determined based only on clinical course and radiological findings; it is possible that we did not exclude other disorders, such as pulmonary infectious disease, from OP. The relatively small cohort size is another limitation. Despite these study limitations, the results of this study contribute to our knowledge of OP after SBRT for lung tumors. Evaluation in large-scale and prospective studies are warranted to confirm our findings.

In conclusion, we have described the characteristics of OP after SBRT for lung tumors. The incidence is relatively high, especially in patients with prior G2/3 RP. The tumor location in the lung might affect the likelihood of development of OP. The latency period for OP is relatively long, and OP could be considered a late adverse event after SBRT. Our findings suggest that long-term and close follow-up is required after SBRT for lung tumors.

## FUNDING

Funding to pay the Open Access publication charges for this article was provided by Matsusaka Central Hospital.
